# Sex Differences in Spontaneous Brain Activity in Adolescents With Conduct Disorder

**DOI:** 10.3389/fpsyg.2018.01598

**Published:** 2018-08-30

**Authors:** Wanyi Cao, Xiaoqiang Sun, Daifeng Dong, Shuqiao Yao, Bingsheng Huang

**Affiliations:** ^1^Medical Psychological Center, The Second Xiangya Hospital, Central South University, Changsha, China; ^2^School of Biomedical Engineering, Health Science Center, Shenzhen University, Shenzhen, China

**Keywords:** conduct disorder, resting-state functional magnetic resonance imaging, amplitude of low-frequency fluctuations, fractional amplitude of low-frequency fluctuations, sex differences, gender paradox hypothesis

## Abstract

**Purpose:** Sex differences in conduct disorder (CD) pathophysiology have yet to be resolved. In this study, we applied the amplitude of low-frequency fluctuations (ALFF) and fractional ALFF (fALFF) to compare spontaneous brain activity in male versus female adolescents diagnosed with CD in light of the gender paradox hypothesis.

**Materials and Methods:** Resting-state functional magnetic resonance imaging (rs-fMRI) examinations were conducted with 51 CD patients (35 males) and 53 age-matched healthy controls (HCs; 35 males). Pearson analysis was conducted to detect relationship between ALFF/fALFF values in gender-differentiated regions and clinical characteristics.

**Results:** We observed that male CD patients showed significant increased ALFF in the bilateral superior temporal gyrus (STG)/insula, and significant decreased ALFF in the left anterior cingulate cortex (ACC), left middle frontal gyrus (BA8 andBA11), left middle temporal gyrus and left inferior/middle temporal gyrus relative to female CD patients. The fALFF in male CD patients was significantly increased in the right STG/insula, decreased in the right superior frontal gyrus, left middle frontal gyrus, right inferior frontal gyrus, and right postcentral gyrus relative to female CD patients. Considering the sex-by-diagnosis interactions in CD patients, the male CD patients had significantly higher fALFF in the left putamen, lower fALFF in the right postcentral gyrus relative to the female CD patients.

**Conclusion:** The brain regions whose activity index values differed in relation to sex should be further explored in CD pathophysiology studies, particularly with respect to sex differences in clinical symptoms, emotional features, cognitive features, and prevalence rates in CD. The present findings are consistent with the gender paradox hypothesis.

## Introduction

Conduct disorder (CD) is a common mental disorder of childhood and adolescence characterized by behaviors that are dismissive of others’ basic rights and societal norms (Segal, 2000). CD may have adverse long-term mental and physical outcomes, including perhaps being a precursor of antisocial personality disorder in adulthood (Kruesi et al., 2004; Michalska et al., 2015). CD can be a serious burden to individuals, families, and society (Scott et al., 2001). CD is diagnosed less often in girls than in boys (incidence, 9.2 vs. 16%) (Loeber et al., 2009) and its symptom presentation is also different in girls than in boys. Notably, girls with CD are more likely to develop relational aggression, as opposed to the physical aggression typical of boys (Kroneman et al., 2009), and more likely to have comorbid mental disorders, such as anxiety and depression (Rosenfield and Mouzon, 2013). Relative to boys diagnosed with CD, girls diagnosed with CD tend to have a greater aggregation of genetic and/or environmental risk factors (Berkout et al., 2011) and may have more severe symptoms, despite a lower prevalence of conduct problems in females (Tiet et al., 2001). These observations fit with the so-called gender paradox hypothesis, which posits that a disorder that has lower occurrence in a particular sex may be associated with more severe symptoms in that sex (Eme, 1992; Loeber and Keenan, 1994). Sex differences in pathogenesis may involve many factors, including genetic, environmental, and psychosocial factors (Sjöberg et al., 2007; Byrd and Manuck, 2014). However, the pathophysiological mechanisms underlying sex differences in CD have not been clarified.

Clarifying sex differences in brain activities is important to gaining a full understanding of antisocial and aggressive behaviors and deciphering the neural mechanisms related to the atypical emotional processing in CD in particular. The majority of fMRI studies have found that the CD adolescents (including males and females) exhibited abnormal activities in insula (Lockwood et al., 2013; White et al., 2013), anterior cingulate cortex (ACC) (Sterzer et al., 2005; Lockwood et al., 2013), amygdala (Sterzer et al., 2005; Cohn et al., 2015), orbitofrontal cortex (Finger et al., 2011), and striatum (Cohn et al., 2015) in cognitive and affective tasks, however, sex differences in CD patients were neither explored nor discussed clearly in these studies. Few imaging studies have investigated sex differences in CD. Fairchild et al. (2013) observed a main effect of CD diagnosis on right amygdala volume in both sexes and a significant sex-by-diagnosis interaction in the bilateral anterior insula. Zhang et al. (2014a) revealed that male CD patients showed higher fractional anisotropy of the bilateral uncinate fasciculus and lower radial diffusivity of the left uncinate fasciculus compared with female CD patients. Michalska et al. (2015) reported that CD symptoms correlate negatively with superior temporal gyrus (STG) gray matter volume, with this correlation being stronger in girls than in boys. Recently, Smaragdi et al. (2017) found that, relative to healthy controls (HCs), both male and female CD patients had thinner cortical thickness and higher gyrification in the ventromedial prefrontal cortex. The variability of these findings might be due to psychiatric comorbidities, sample heterogeneity, or differing analysis methods.

Resting-state functional magnetic resonance imaging (rs-fMRI) is a reliable neuroimaging technique in which subjects keep their eyes either closed or fixated on a crosshair during scanning in a task-free paradigm. In rs-fMRI, low frequency (0.01–0.08 Hz) fluctuations in blood oxygen level-dependent (BOLD) signals during rest are highly associated with spontaneous neuronal activity (Biswal et al., 1995). The amplitude of low-frequency fluctuations (ALFF) represents the absolute intensity within the low-frequency range (Zang et al., 2007), while fractional ALFF (fALFF) reflects the ratio of power of low-frequency band to that of detectable frequency range (Zou et al., 2008). ALFF has slightly higher test–retest reliability whereas fALFF has higher specificity in gray matter regions (Zuo et al., 2010). The selecting of two measurements was to maximize the reliability of our study. The ALFF and fALFF analysis have been broadly used in studies of various psychiatric and behavioral disorders, including schizophrenia (Hoptman et al., 2010), attention-deficit/hyperactivity disorder (Yang et al., 2011), and major depressive disorder (Guo et al., 2012; Liu et al., 2013).

To our knowledge, no previously published rs-fMRI studies of CD have included female subjects (Lu et al., 2015, 2017; Zhou et al., 2015, 2016; Pu et al., 2017; Wu et al., 2017; Zhang et al., 2017), let alone explored rs-fMRI gender differences in CD. In this study, we applied ALFF and fALFF to compare spontaneous brain activity between males and females with CD. We hypothesized that there would be significant gender-related differences in ALFF/fALFF in CD patients that may support the gender paradox hypothesis in CD.

## Materials and Methods

### Participants

Adolescents with CD (35 males and 16 females, mean age = 14.69 years, *SD* = 0.76) were recruited from out-patient clinics affiliated with the Second Xiangya Hospital of Central South University (Changsha, Hunan, China). A group of 53 gender- and age-matched HCs (35 males and 18 females, mean age = 14.83 years, *SD* = 0.51) were recruited from middle schools in Changsha. All participants were aged between 12 and 17 years old.

Diagnosis of CD was made by two well-trained psychiatrists based on the Structural Clinical Interview for DSM-IV-TR Axis I Disorder-Patient Edition (SCID-I/P) (First et al., 2001). Detailed information about each patient was obtained from a parent. If the information provided by patients was inconsistent with that obtained by their parents, the psychiatrists made a final judgment.

For all participants, the exclusion criteria were a history of attention-deficit/hyperactivity disorder, any psychiatric or emotional disorder (e.g., post-traumatic stress disorder), any pervasive developmental disorder (e.g., autism spectrum disorder), any chronic neurological disorder (e.g., Tourette’s syndrome, obsessive compulsive disorder), persistent headaches, head trauma, alcohol/substance abuse in the past year, contraindications to magnetic resonance imaging, or an intelligence quotient (IQ) ≤ 80 on the Wechsler Intelligence Scale for Children-Chinese revision (C-WISC) examination (Gong and Cai, 1993). All CD patients were adolescent-onset CD who exhibited CD symptoms after 10 years old (Segal, 2000). All participants were right-handed, as determined by the Edinburgh Handedness Inventory (Oldfield, 1971). None of the HC volunteers met the SCID-I/P diagnostic criteria for CD or any other psychiatric disorders.

The study was approved by each school’s administration and the Ethics Committee of the Second Xiangya Hospital of Central South University. All subjects and their parents were informed of the study’s goal and the participant’s parents provided written informed consent.

### Self-Report Assessments

All participants completed the Barratt Impulsivity Scale-version 11 (BIS-11), including three subscales attention impulsiveness (attention and cognitive instability dimensions), non-planning impulsiveness (self-control and cognitive complexity dimensions), motor impulsiveness (motor and perseverance dimensions) (Yao et al., 2007), and the Antisocial Process Screening Device (APSD), to evaluate callous-unemotional (CU) trait levels (Vitacco et al., 2003). These scales have showed adequate reliability and validity in previous CD studies (Zhang et al., 2014b; Jiang et al., 2015; Dong et al., 2017; Pu et al., 2017).

### Imaging Data Acquisition

All participants underwent magnetic resonance imaging in a Philips, Achieva, 3.0-T scanner. The functional images were acquired with an echo-planar imaging (EPI) sequence (repetition time/echo time = 2000/30 ms, flip angle = 90°, field of view = 240 mm × 240 mm, matrix = 64 pixels × 64 pixels, thickness/gap = 4.0/0 mm, number of volumes = 206, resting acquisition time = 6 min 52 s). Noise and head movement were reduced by fitting ear plugs and padding around the head.

### Data Preprocessing

Data preprocessing was conducted with Data Processing Assistant for Resting-State fMRI (DPARSF^1^) (Yan and Zang, 2010). The first ten time points for each individual were removed to alleviate the effects of signal instability and participant adaption. Slice timing correction was applied to the middle slice as the reference slice. To reduce motion-related artifacts, realignment for head motion correction and the Friston 24-parameter model were conducted as additional nuisance covariates. The corrected images were normalized to an EPI template at a voxel size of 3 mm × 3 mm × 3 mm, which EPI has become a highly reliable method of normalization and been broadly used in many fMRI studies (Zhu et al., 2014; Michalska et al., 2016; Wu et al., 2017). Spatial smoothing was performed with a Gaussian kernel (4 mm, full-width at half-maximum) and the linear trends were removed. Participants whose head motion exceeded 2.5 mm of translation or 2.5° of rotation in any direction were excluded. We calculated mean frame-wise displacement (FD) for four groups (Power et al., 2012), there was no difference in the mean FD among four groups (**Table 1**). Meanwhile, we excluded the subjects whose head motion exceeded “mean FD + 2SD” (Satterthwaite et al., 2013). Finally, four subjects were removed.

**Table 1:** Characteristic comparisons for male and female CD and HC groups.

Variable	Group, mean ± SD				*F-*value (*p-value)*		
	CD		HC		Sex effect	Dx effect	Sex × Dx
	Male	Female	Male	Female			
Age, years	14.74 ± 0.82	14.56 ± 0.63	14.83 ± 0.57	14.83 ± 0.38	0.418 (*0.519)*	1.724 (*0.192)*	0.465 (*0*.*497)*
C-WISC	101.08 ± 7.52	98.04 ± 8.05	108.92 ± 6.14	107.96 ± 5.86	1.921 (*0*.*169)*	37.792 (*<0.001*^∗∗^)	0.514 (*0*.*475)*
FD	0.22 ± 0.09	0.17 ± 0.07	0.22 ± 0.08	0.21 ± 0.08	1.894 (*0.172)*	0.998 (*0*.*320)*	0.904 (*0*.*344)*
BIS total	74.14 ± 8.82	81.69 ± 10.83	66.29 ± 6.06	65.44 ± 10.88	3.347 (*0.070)*	43.261 (*<0.001*^∗∗^)	5.238 (*0*.*024*^∗^)
BIS-attention impulsivity	18.86 ± 3.07	20.81 ± 4.34	17.34 ± 2.33	16.94 ± 2.65	1.528 (*0.219)*	18.260 (*<0.001*^∗∗^)	3.492 (*0*.*065)*
BIS-motor impulsivity	26.69 ± 5.41	29.44 ± 4.69	21.91 ± 3.90	22.72 ± 4.71	3.258 (*0*.*074)*	33.927 (*<0.001*^∗∗^)	0.972 (*0*.*327)*
BIS-unplanned impulsivity	28.60 ± 5.25	31.44 ± 6.31	27.03 ± 3.36	25.78 ± 5.95	0.570 (*0.452)*	11.842 (*0.001^∗∗^*)	3.785 (*0*.*055*)
APSD-CU trait	5.83 ± 2.22	6.38 ± 2.00	4.40 ± 1.80	3.72 ± 1.64	0.026 (*0.873)*	24.842 (*<0.001*^∗∗^)	2.235 (*0*.*138)*

*CD, conduct disorder; HC, healthy control; SD, standard deviation; Dx, diagnosis; C-WISC, Chinese Wechsler Intelligence Scale for Children; FD, frame-wise displacement; BIS, Barratt Impulsiveness Scale; APSD-CU, Antisocial Process Screening Device callous-unemotional. ^∗^p < 0.05; ^∗∗^p < 0.001.*

### ALFF and fALFF Analysis

Amplitude of low-frequency fluctuations and fALFF analysis were conducted in DPARSF. After the above preprocessing, the fMRI data were temporally band-pass filtered (0.01–0.08 Hz) to remove physiological high-frequency noise and low-frequency drift. The time series of each voxel was converted into the frequency domain, and its power spectrum was calculated. The square root was calculated at each frequency of the power spectrum, and the average square root was then obtained at each voxel across the frequency range of 0.01–0.08 Hz (Zang et al., 2007). The average square root values obtained were taken as ALFF and deemed to reflect absolute intensity of brain spontaneous neural activity (Zang et al., 2007). For the fALFF analysis, the power of the amplitude averaged across 0.01–0.08 HZ was divided by that the entire frequency range (0–0.25 Hz) at each voxel (Zou et al., 2008). Brodmann areas (BAs) are designated for clarity.

### Statistical Analysis

Demographic and clinical characteristics were compared between groups with two-way analyses of variance (ANOVAs), with sex and diagnosis as between-group factors, in Statistical Package for Social Sciences for Windows 16.0 (SPSS Inc., Chicago, IL, United States) (Villers Ruiz, 1981). Full-factorial ANOVA analysis in SPM8^2^ was used to investigate main effects of diagnosis, sex, and sex-by-diagnosis interactions based on ALFF and fALFF values with sex and diagnosis as factors and IQ and age as covariates. We defined the regions that showed significant differences in sex effects and sex-by-diagnosis interactions as potential regions of interest, and the *post hoc t*-tests were used to detect the significantly different regions between female and male CD patients. All significance thresholds were set to *p* < 0.05 with false discovery rate correction and a 10-voxel extension threshold.

Pearson correlation was conducted in SPSS 16.0 to explore associations between ALFF and fALFF values in gender-differentiated regions and clinical characteristics in CD patients subgroups separately. For multiple comparison, a Bonferroni correction was conducted.

## Results

### Demographic and Clinical Characteristics

The demographic and clinical characteristics of the four groups are summarized in **Table 1**. The four groups were well-matched in terms of age and FD. There were no significant differences in demographic or clinical characteristics between females and males. Relative to HCs, subjects with CD had a significantly lower mean IQ scores and had higher mean impulsivity (i.e., BIS-11) and CU trait (i.e., APSD-CU) scores. Female CD patients had higher impulsivity and CU trait scores than the other three groups.

### ALFF

**FIGURE 1:**
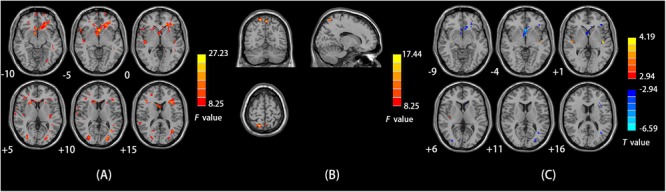
**(A)** Brain regions showing sex effects on ALFF among CD patients and HCs. **(B)** Brain regions showing a sex-by-diagnosis interaction for ALFF differences among four groups. **(C)** Brain regions showing differences in ALFF between males and females with CD by sex effects. Warm and cool colors, respectively, indicate brain regions with increased and decreased ALFF in male vs. female CD patients. For all panels, the effect criterion for view was uncorrected *p* < 0.005. The left of figure correspond to the right of brain.

We observed a main effect of sex (female vs. male) on ALFF in the left middle frontal gyrus, left superior frontal gyrus, right inferior frontal gyrus, right STG, left middle temporal gyrus, left middle occipital gyrus, left parahippocampal gyrus, and left cerebellum posterior lobe (**Figure 1A**). Additionally, we observed a main effect of diagnosis (CD vs. HC) on ALFF in the left insula, right precuneus, left lingual gyrus, and left middle frontal gyrus. There was a sex-by-diagnosis interaction of ALFF in the right superior parietal lobule/precuneus (**Figure 1B**). Regarding the main effects of sex, relative to the female CD group, the male CD group had higher ALFF in the bilateral STG/insula and lower ALFF in the left ACC, left middle frontal gyrus (BA8 and BA11), left middle temporal gyrus, and left inferior/middle temporal gyrus (**Figure 1C** and **Table 2**). Regarding the sex-by-diagnosis interaction, there was no significantly different regions in male CD and female CD patients.

**Table 2:** Brain areas exhibiting significant ALFF differences between male and female CD patients.

Brain region	Cluster size, voxels	MNI coordinates			Peak *t*-value
		X	Y	Z	
***Main effect of sex***					
*CD Male > CD Female*					
L STG/insula	12	–42	–15	0	4.17
R STG/insula	14	48	–18	3	4.19
*CD Male < CD Female*					
L ACC	106	0	18	–6	–6.59
L middle frontal gyrus (BA 11)	25	–21	45	–12	–4.03
L middle frontal gyrus (BA 8)	29	–21	27	36	–4.51
L middle temporal gyrus	11	–36	–60	15	–4.58
L inferior/ middle temporal gyrus	13	–45	–24	–15	–4.32

*ALFF, amplitude of low-frequency fluctuations; CD, conduct disorder; MNI, Montreal Neurological Institute; L, left; R, right; STG, superior temporal gyrus; ACC, anterior cingulate cortex; BA, Brodmann area. All p < 0.05 with false discovery rate correction and a 10-voxel extension threshold.*

### fALFF

**FIGURE 2:**
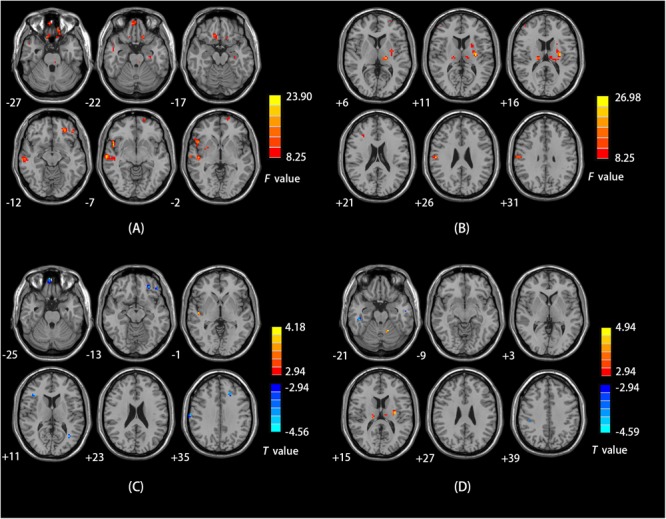
**(A)** Brain regions showing sex effects on fALFF among CD patients and HCs. **(B)** Brain regions showing a sex-by-diagnosis interaction for fALFF differences among four groups. **(C)** Brain regions showing differences in fALFF between males and females with CD by sex effects. **(D)** Brain regions showing differences in fALFF between males and females with CD by sex-by-diagnosis interaction effects. For both **C,D**, Warm and cool colors, respectively, indicate brain regions with increased and decreased fALFF in male vs. female CD patients. For all panels, the effect criterion for view was uncorrected *p* < 0.005. The left of figure correspond to the right of brain.

**FIGURE 3:**
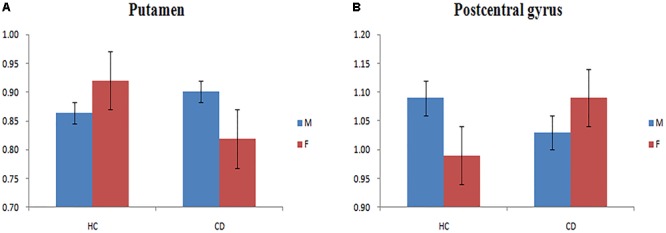
**(A)** Bar chart showing a sex-by-diagnosis interaction for fALFF differences among four groups in the left putamen. **(B)** Bar chart showing a sex-by-diagnosis interaction for fALFF differences among four groups in the right postcentral gyrus.

We observed a main effect of sex (female vs. male) on fALFF in the bilateral middle frontal gyrus, bilateral inferior frontal gyrus, right middle temporal gyrus, right temporal lobe, right precentral gyrus (**Figure 2A**). Additionally, we observed a main effect of diagnosis (CD vs. HC) on fALFF in the left insula, left precentral gyrus, right cerebellum posterior lobe, left inferior parietal lobule, and left precuneus. There was a sex-by-diagnosis interaction of fALFF in the left thalamus, left putamen, and right postcentral gyrus (**Figure 2B**). Regarding the main effects of sex, relative to the female CD group, the male CD group had higher fALFF in the right STG/insula and lower fALFF in the right superior frontal gyrus, left middle frontal gyrus, right inferior frontal gyrus, right postcentral gyrus (**Figure 2C** and **Table 3**). Regarding the sex-by-diagnosis interaction, relative to the female CD group, the male CD group had higher fALFF in the left putamen, lower fALFF in the right postcentral gyrus (**Figures 2D, 3** and **Table 3**).

**Table 3:** Brain areas exhibiting significant fALFF differences between male and female CD patients.

Brain region	Cluster size, voxels	MNI coordinates			Peak *t*-value
		X	Y	Z	
***Main effect of sex***					
*CD Male > CD Female*					
R STG/insula	16	48	–15	0	4.18
*CD Male < CD Female*					
R superior frontal gyrus	12	6	60	–24	–4.03
L middle frontal gyrus (BA 11)	10	–27	42	–12	–4.41
L middle frontal gyrus	15	–18	30	36	–4.22
R inferior frontal gyrus	10	39	27	12	–4.56
R postcentral gyrus	20	66	–15	33	–4.49
***Sex-by-diagnosis interaction***					
*CD Male > CD Female*					
L putamen	37	–30	–15	12	4.55
*CD Male < CD Female*					
R postcentral gyrus	12	42	–30	36	–4.12

*fALFF, fractional amplitude of low-frequency fluctuations; CD, conduct disorder; MNI, Montreal Neurological Institute; L, left; R, right; STG, superior temporal gyrus; BA, Brodmann area. All p < 0.05 with false discovery rate correction and a 10-voxel extension threshold.*

### Correlation Analysis

No significant correlation results were detected between ALFF and fALFF values in gender-differentiated regions and BIS-11 scores and CU traits in CD patients subgroups after Bonferroni correction.

## Discussion

To our knowledge, this is the first rs-fMRI study to use both ALFF and fALFF to investigate the influence of gender on spontaneous brain activity in CD patients. We obtained evidence of gender effects on CD-related abnormal brain activity. Regarding the main effects of sex, relative to the female CD patients, male CD patients had higher ALFF and fALFF values in the bilateral STG/insula and lower ALFF and fALFF values in the left ACC, right superior frontal gyrus, right inferior frontal gyrus, left middle frontal gyrus (BA8 and BA11), left middle temporal gyrus, left inferior/middle temporal gyrus, and right postcentral gyrus. Regarding the sex-by-diagnosis interactions, the fALFF in male CD patients was significant increased in the left putamen and decreased in the right postcentral gyrus relative to female CD patients.

Our finding of lower ALFF and fALFF in the bilateral STG of female, relative to male, CD patients should be considered in the context of prior STG findings in CD patients. Structural deficits in the STG have been reported in both males (Fairchild et al., 2015) and females (Budhiraja et al., 2017) with CD. A voxel-based morphometry study showed a stronger negative correlation between CD symptoms and STG gray matter volume in girls than in boys (Michalska et al., 2015). In a recent rs-fMRI study conducted in a study population of all male CD patients, Zhou et al. (2016) observed abnormally decreased default mode network (DMN) activity in the STG. In another recent fMRI study, Michalska et al. (2016) observed a sex difference in the STG of children with CD symptoms performing an others’ harm task. The right STG, which is a component of the human ventral attention system (Corbetta et al., 2008), plays an important role in social cognition and perception processes (Pelphrey et al., 2004) and facial emotion recognition (Allison et al., 2000). Abnormal STG activity during emotional and neutral facial expression processing might be relevant to clinical features of antisocial behavior in CD patients (Passamonti et al., 2010). Sex differences in the STG may be related to differential expression of antisocial behaviors in female versus male adolescents with CD.

Relative to the male CD patients, the female CD patients in our study had increased ALFF in the left ACC. Functionally, the ACC has been associated with the processing social information (Behrens et al., 2008) and reward-related information (Lockwood et al., 2015) as well as with empathy of pain processing (Marsh et al., 2013) and emotion regulation (Shackman et al., 2011). A quantitative meta-analysis of a sample of 266 boys with disruptive behavior disorders pointed to significant structural and functional abnormalities in the ACC (Gavita et al., 2012). Another structural neuroimaging study found that female CD patients had reduced gray matter volumes in the ACC compared with female HCs (Budhiraja et al., 2017). In a study employing an affective stimulation task, ACC activation was found to be related to novelty seeking, a core temperamental trait related to impulsivity and a quick-tempered personality, as well as to disadvantageous cognitive control strategies in CD patients (Stadler et al., 2007). The interpretation of the findings in the above studies was that an abnormal ACC may contribute to aggressive behavior, impulsivity, and emotion imbalance in CD.

Our findings of higher impulsivity and CU trait scores in females with CD (the lower incidence gender) than in males with CD are consistent with the gender paradox hypothesis. Current findings implied that gender differences in ACC among CD patients may be associated with higher impulsivity and CU trait levels in female CD patients than in male CD patients. Our findings underscore the importance of exploring this region’s structure and function in relation to gender in CD patient populations.

Female CD patients in our study also showed increased ALFF and fALFF in the right superior frontal gyrus, right inferior frontal gyrus, left middle frontal gyrus, and left inferior/middle temporal gyrus, relative to those observed for male CD patients. Previous rs-fMRI studies have reported decreased activity in the MFG and middle temporal gyrus in CD patients relative to activity seen in HCs (Dalwani et al., 2014; Lu et al., 2015; Wu et al., 2017). The pattern of sex differences in the MFG in our study was consistent with previous findings of sex differences in the MFG in children with conduct problems performing affective tasks (Michalska et al., 2016). The frontal lobe plays an important role in high-level executive function and emotion regulation (Chayer and Freedman, 2001). The middle temporal gyrus has been implicated in visual social signals and social cognitive systems (Sugiura et al., 2013). Differences in frontal gyrus and middle temporal gyrus in the CD patients of different genders might explain, at least in part, differing emotional and cognitive behaviors in male and female CD patients.

Considering the sex-by-diagnosis interactions in CD patients, the fALFF in female CD patients was significant increased in the right postcentral gyrus and decreased in the left putamen relative to male CD patients, but the opposite pattern of sexual differentiation in the two regions existed in typical population. The postcentral gyrus is the primary somatosensory cortex, located in lateral parietal lobe (Kaas and Collins, 2001). The putamen plays an important role in processing facial expressions of disgust (Phillips et al., 1998; Sprengelmeyer et al., 1998). Prior rs-fMRI studies have demonstrated abnormal activity in the right postcentral gyrus and left putamen in male CD patients (Lu et al., 2015; Korponay et al., 2017). The sex differences in the regions associated with low-order cognitive processing and emotion recognition between male and female CD patients may help to explain the higher CD diagnosis rates among males than among females. The presently observed opposite pattern of sex differences in CD versus HCs in the right postcentral gyrus and left putamen may be related to particular neurodevelopmental mechanism of CD. These results require further confirmation and exploration in future studies.

The current study had several limitations. First, the small sample size of females with CD may limit the generalizability of the current results. Larger CD samples are needed to confirm the presently reported sex differences. Second, because both of our CD groups, male and female, had lower IQ scores than the two HC groups, IQ scores were not matched between CD patients and HCs in the main analysis. To reduce the effect of IQ on our results, we included IQ as a covariate in the analyses. Third, the study did not discuss early-onset CD samples, so whether the observed sex differences in spontaneous brain activity were limited in adolescent-onset CD needs to be explored. Finally, in the future, multi-parametric neuroimaging studies with larger, more diverse samples should be used to explore the pathophysiology of sex differences in CD.

## Conclusion

The present rs-fMRI findings provide insights into gender differences in the pathophysiology of CD. Using ALFF and fALFF as indexes of spontaneous neuronal activity, we observed resting-state activity differences between male and female CD patients in frontal lobe, temporal lobe, left ACC, right postcentral gyrus, and left putamen. These regions have been associated with clinical symptoms, emotion, and cognition. Elucidating these regions’ gender-associated differences in CD may be useful for resolving the reasons underlying gender differences in CD diagnosis prevalence rates. The present findings are consistent with the gender paradox hypothesis.

## Author Contributions

SY and BH conceived and designed the study. WC, XS and DD collected and analyzed the data. XS and DD helped to draft the manuscript. WC wrote the manuscript.

## Conflict of Interest Statement

The authors declare that the research was conducted in the absence of any commercial or financial relationships that could be construed as a potential conflict of interest.

## Footnotes

1 http://rfmri.org

2 http://www.fil.ion.ucl.ac.uk/spm
